# A pilot observational study on magnesium and calcium imbalance in elderly patients with acute aortic dissection

**DOI:** 10.1186/s12979-016-0083-y

**Published:** 2017-01-05

**Authors:** E. Vianello, E. Dozio, A. Barassi, G. Sammarco, L. Tacchini, M. M. Marrocco-Trischitta, S. Trimarchi, M. M. Corsi Romanelli

**Affiliations:** 1 Department of Biomedical Sciences for Health, Chair of Clinical Pathology, Università degli Studi di Milano, via Luigi Mangiagalli 31, 20133 Milan, Italy; 2 Department of Health Sciences, Università degli Studi di Milano, Milan, Italy; 3 Laboratory Medicine Operative Unit-1, Clinical Pathology, I.R.C.C.S. Policlinico San Donato Milanese, Milan, Italy; 4 Thoracic Aortic Research Center, I.R.C.C.S. Policlinico San Donato, San Donato Milanese, Milan, Italy

**Keywords:** Magnesium (Mg), Calcium (Ca), Acute aortic dissection (AAD)

## Abstract

**Background:**

Magnesium (Mg) and calcium (Ca) are the principal essential elements involved in endothelial cell homeostasis. Extracellular changes in the levels of either alter endothelial contraction and dilatation. Consequently Mg and Ca imbalance is associated with a high risk of endothelial dysfunction, the main process observed during acute aortic dissection (AAD); in this clinical condition, which mainly affects elderly men, smooth muscle cell alterations lead to intimal tears, creating a false new *lumen* in the *media* of the aorta. AAD patients have a high risk of mortality as a result of late diagnosis because often it is not distinguished from other cardiovascular diseases.

We investigated Mg and Ca total circulating levels and the associated pro-inflammatory mediators in elderly AAD patients, to gain further information on the pathophysiology of this disorder, with a view to suggesting newer and earlier potential biomarkers of AAD.

**Results:**

Total circulating Mg and Ca levels were both lower in AAD patients than controls (*p* < 0.0001). Using Ca as cut-off, 90% of AAD patients with low Ca (<8.4 mg/dL) came into the type A classification of AAD. Stratifying AAD according to this cut-off, Mg was lower in patients with lower total Ca.

Compared to controls, both type A and B AAD patients had higher levels of all the pro-coagulant and pro-inflammatory mediators analyzed, including sP-sel, D-dimer, TNF-α, IL-6, and CRP (*p* < 0.05). Dividing types A and B using the Stanford classification, no significant differences were found (*p* > 0.05) The levels of both ICAM-1 and EN-1 were lower in AAD than in a control group (*p* < 0.0001 and *p* < 0.05 respectively).

**Conclusions:**

These findings suggest that low Mg and Ca in AAD elderly patients may contribute to altering normal endothelial physiology and also concur in changing the normal concentrations of different mediators involved in vasodilatation and constriction, associated with AAD onset and severity.

## Background

Magnesium (Mg) and calcium (Ca) are involved in the most essential processes regulating cardiovascular function and their imbalance is a factor in the development of numerous disorders of the cardiovascular system, mostly linked to endothelial dysfunction and inflammation, mainly affecting vascular smooth muscle cells (VSMCs) [1]. In humans and animals extracellular Mg has cardioprotective properties because it attenuates all the agonist-induced vasoconstriction molecules, including endothelin-1 (EN-1), helping preserve vascular tone and preventing coronary vasospasm [1–5]. These cardioprotective effects are reinforced by the fact that Mg assists the coagulation reactions, binding specific coagulation proteins in case of endothelial damage [6].

In the cardiovascular system, one of the main mechanisms that regulates VSMC activity during pressure load is the Ca channel block mediated by Mg [7–14]. This implies that Mg depletion is associated with a high risk of cardiovascular disease (CVD) [2] but little is known about the Mg/Ca concentrations in acute aortic dissection (AAD), a dramatic cardiovascular disorder that is often fatal because of late diagnosis [15]. The etiology of AAD involves either genetic disorders affecting connective tissue, such as Marfan syndrome, or is a consequence of primary disorders, generally called non-Marfan AAD, both of which involve aortic stiffening, reduced coronary vessel flow, increased pulse pressure and left ventricular dysfunction [1, 15, 16].

Aging is one of the main risk factors leading to AAD: these include atherosclerosis, hypertension and calcification of the endothelial tunica [17–19]. In particular, patients younger than 65 years old with AAD, undergoing coronary bypass surgery, showed active formation of calcification in atherosclerotic plaques [16, 18, 19].

A discrete and temporary intracellular Ca increase, due to Na/K pump inhibition or blocking a natural Ca antagonist like Mg, may alter cell contraction and transcription and cause cell death [20]. This pathophysiological effect may evolve, at endothelial level, in pro-inflammatory mechanisms characteristic of AAD, including the release of pro-inflammatory mediators, pro-coagulant and endothelial factors from damaged vascular cells [1, 21, 22].

Our aim was to record total circulating Mg and Ca levels in AAD, looking for their possible implication in the severity of the disorder, which is also characterized by the release of mediators involved in endothelial dysfunction, including endothelial, pro-inflammatory and pro-coagulant factors.

## Methods

### Patients

At I.R.C.C.S. Policlinico San Donato in Milan we enrolled 33 males (age 40–86 years, mean 62.2 ± 18.6 years) with only non-Marfan AAD to exclude any genetic confounding factors, diagnosed within 24 h of symptom onset; 30 healthy age-matched individuals were enrolled as a control group. AAD patients were included in the International Registry of Aortic Dissection (IRAD) that was set up in 1996 by cardiovascular specialists committed to expanding current knowledge of aortic dissection with the goal of improving patient outcomes, with data from 1 January 1996 to 2015. The structure and methods of IRAD have been published [23, 24] and patients gave the hospital their written informed consent.

Patients were then classified according to the Stanford classification in two groups: type A, comprising 22 patients in whom the dissection involved the ascending aorta (proximal dissection) and type B, comprising 11 in whom the dissection was limited to the descending aorta (distal dissection). The type AAD in-patients had no prior aortic dissection and no prior mitral, bicuspid, cuspid or aortic valve diseases. All patients were non-smokers and had never used a drug of abuse (cocaine).

### Assays

Serum and plasma were separated by 15 min centrifugation at 1000 g, and stored frozen at −20 °C until analysis. Total Mg was determined by a colorimetric method based on xylidyl blue reaction in alkaline medium to form a water-soluble purple-red chelate whose color intensity is proportional to the concentration of Mg ions in the sample. Calcium ion is excluded from the reaction by complexing with EGTA. For detection we used an RX Monza spectrophotometer (Randox Laboratories, Crumlin, County Antrim, United Kingdom) according to the manufacturer’s protocol, with a clinical cut-off from 1.7 to 2.1 mg/dL. Intra- and inter-assay precision was respectively 0.70 and 2.71% (coefficient of variation - CV%). Total Ca was also determined using a colorimetric method with Vitros 5600 (VITROS Chemistry Products Ca Slides, Ortho Clinical Diagnostics, Rochester), and the clinical values ranged between 8.4 and 10.2 mg/dL. The CV% was 0.04 to 0.12%.

Human soluble P-selectin (s-Psel), interleukin-6 (IL-6), tumor necrosis factor-α (TNF-α), interleukin-1β (IL-1β), coagulation factor III (CFIII), endothelin-1 (EN-1), and intercellular adhesion molecule 1 (ICAM-1) were measured by enzyme-linked immunosorbent assays (ELISA) according to the manufacturer’s directions (Quantakine Immunoassay, R&D System, Minneapolis, Minnesota, USA). Intra- and inter-assay precision (% CV) was as follows: s-Psel intra-assay mean precision 5.2% and inter-assay mean precision 8.86%; IL-6 intra-assay mean precision 2.6% and inter-assay mean precision 4.5%; TNF-α intra-assay mean precision 4.9% and inter-assay mean precision 7.6%; IL-1β intra-assay mean precision 5.4% and inter-assay mean precision 5.6%; CFIII intra-assay mean precision 2.83% and inter-assay mean precision 5.8%, EN-1 intra-assay mean precision 2.73% and inter-assay mean precision 6.26%, ICAM-1 intra-assay mean precision 4.63% and inter-assay mean precision 5.3%.

Human high-sensitive C-reactive protein (hCRP) was assayed by immunonephelometry (BN II, Dade Behring, Marburg, Germany) with intra- and inter-assay CV% less than 8 and 6% respectively. D-dimer was quantified by immunoreactions using Cobas® 6000 platform analyzer series (Roche Diagnostics, Milan, Italy) with intra- and inter-assay CV% 1.7 and 3.1%.

### Statistical analysis

Data were expressed as mean ± standard deviation (SD) and analyzed using the GraphPad Prism 6.0 biochemical statistical package (GraphPad Software, Inc., San Diego, CA). The normality of data distribution was assessed by the Kolmogorov-Smirnoff test. Groups were compared using Student’s two-tailed unpaired *T*-test or the Mann–Whitney *U*-test, as appropriate. Correlations between parameters were evaluated using the Spearman test. A *p* value <0.05 was considered statistically significant.

## Results

**Fig. 1:**
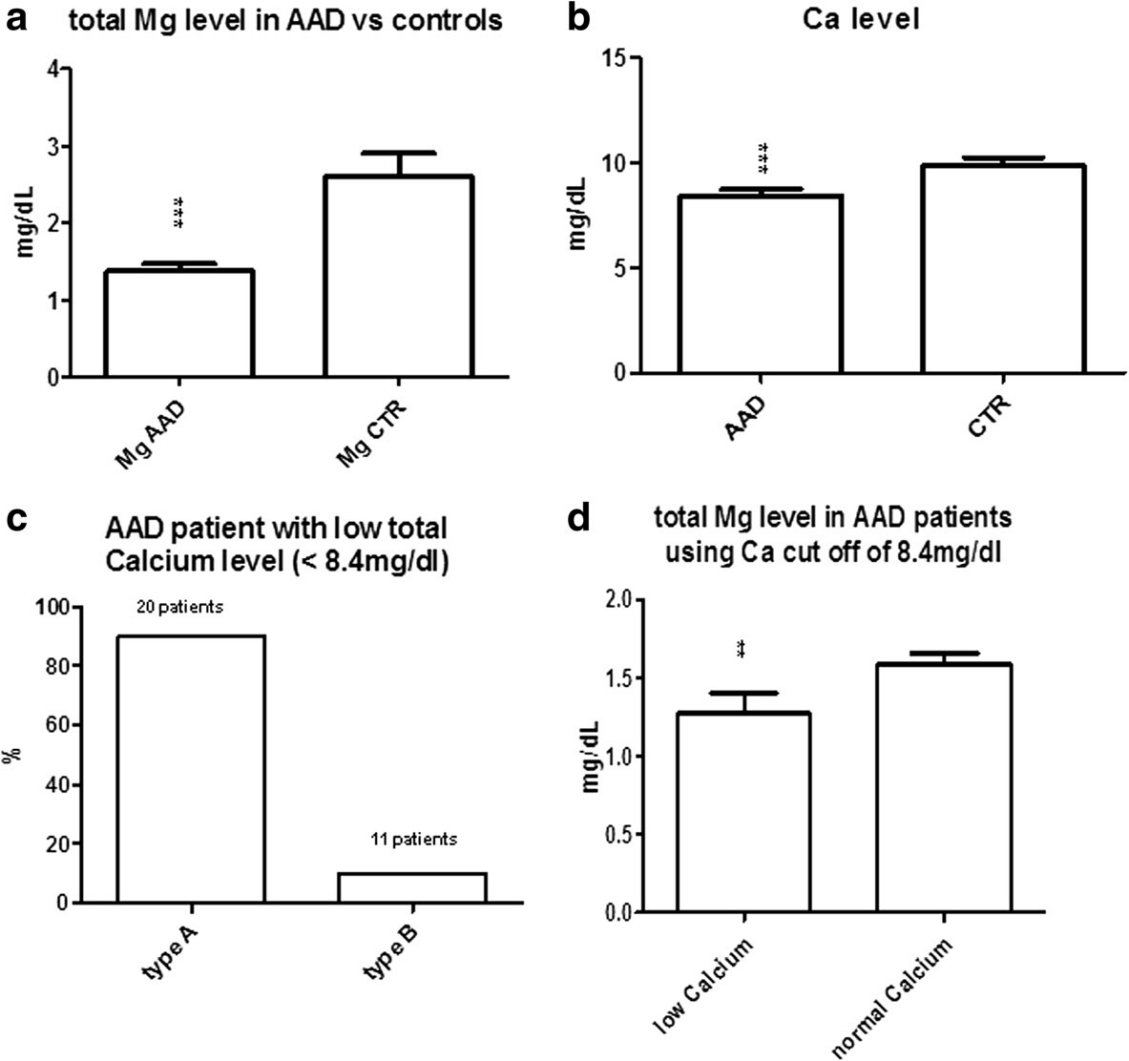
Total Mg and Ca levels in AAD patients. **a** AAD patients had significantly lower total Mg than controls (*p* < 0.0001). **b** AAD patients had significantly lower total Ca than controls. **c** 90% of AAD patients with Ca below the lower Ca cut-off of 8.4 mg/dL were type A of the Stanford classification. **d** Stratifying patients on the basis of the lower clinical Ca cut-off, Mg was lower in AAD patients with low total Ca (*p* < 0.01)

Circulating Mg and Ca levels were significantly lower in AAD patients than in the control group (*p* < 0.0001) (Fig. 1a–b) but no significant correlation was found between the two parameters (data not shown). Using a clinical cut-off for Ca of 8.4–10.2 mg/dL, 90% of AAD patients with low total Ca (<8.4 mg/dL) were classified as type A (Fig. 1c). Dividing the patients using the 8.4 mg/dL Ca cut-off, total circulating Mg was significantly lower in patients with low total calcium (*p* < 0.01) (Fig. 1d).

**Fig. 2:**
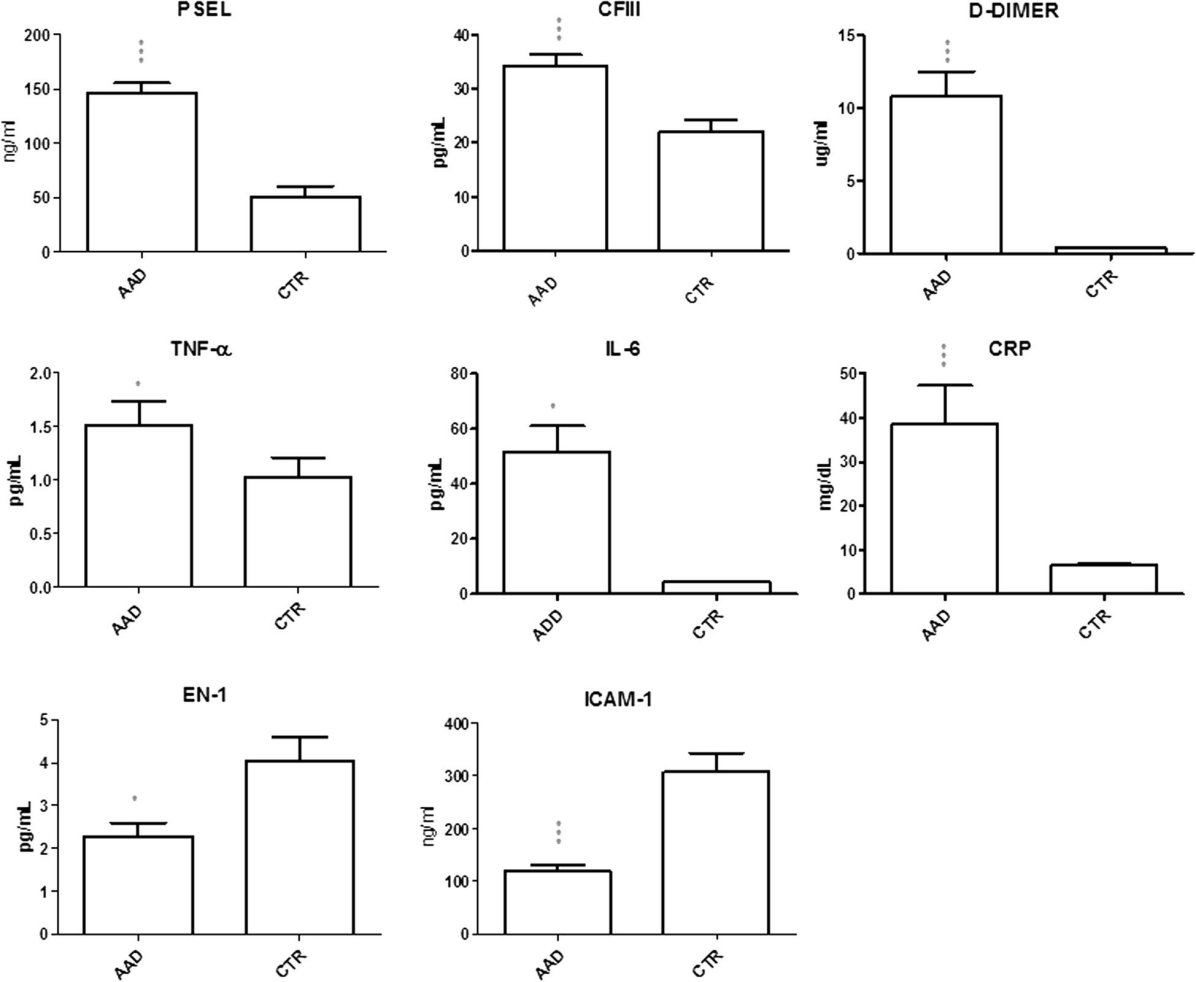
Pro-coagulant, pro-inflammatory and endothelial mediator levels in AAD patients and controls. AAD patients had higher levels of pro-coagulant mediators including CFIII, sP-sel and D-dimer (all *p* < 0.0001). Patients also had higher levels of pro-inflammatory mediators including TNF-α (*p* < 0.05), IL-6 (*p* < 0.05) and CRP (*p* < 0.0001) than controls. The endothelial mediators ICAM-1 and EN-1 were significantly lower in AAD patients than controls (*p* < 0.0001 and *p* < 0.05 respectively)

Compared to controls, types A and B AAD patients all had higher levels of all pro-coagulant mediators including CFIII, s-Psel, and D-dimer (*p* < 0.0001 for all) (Fig. 2). Human TNF-α, IL-6, and CRP, as pro-inflammatory mediators, were higher in the overall AAD group than controls (*p* < 0.05, *p* < 0.05 and *p* < 0.0001 respectively) (Fig. 2). Dividing type A and B using the Stanford classification, no significant differences were seen in either group.

The endothelial mediators analyzed, including ICAM-1 and EN-1, were lower in AAD patients than controls (*p* < 0.0001 and *p* < 0.05 respectively) (Fig. 2). No significant differences were found between type A and B patients (*p* > 0.05).

No significant correlations were found between Mg and Ca circulating levels and the levels of any of the pro-inflammatory, pro-coagulator and endothelial parameters (data not shown).

## Discussion

This study focusing on AAD found low total magnesemia and calcemia. Mg total circulating levels were markedly lower in AAD patients than controls (Fig. 1a) and the pattern was similar for total Ca (Fig. 1b). Because Mg is the natural antagonist of Ca and both these elements have a primary role in VSM tone [14], we also compared the Mg concentration with total Ca. On stratifying AAD patients on the basis of the lower total Ca cut-off (clinically established as less than 8.4 mg/dL), 90% were type A, which is recognized as having a worse prognosis and higher risk of death than type B (Fig. 1c) [17]. These outcomes suggest that in AAD patients the extracellular Mg deficiency, known as an indicator of severe intracellular Mg depletion, directly influences total Ca. Therefore we assume that the lower extracellular Mg levels in AAD patients were too low to balance Ca entry into the cells, resulting in a total circulating Ca deficiency.

On the basis of this preliminary data we can only suggest that during the acute phase of AAD, within 24 h, the molecular mechanism may be driven by Mg/Ca deficiency promoting an imbalance among the concentrations of several mediators involved in inflammation and vasoconstriction of the endothelium. We therefore measured the circulating levels of the main pro-inflammatory mediators that can be directly affected by Mg/Ca and by endothelial dysfunction, including TNF-α, IL-6 and CRP [25–27]: they were all substantially higher in AAD patients than controls. The systemic inflammation highlighted in AAD patients was also associated with an imbalance of pro-coagulant and vasoconstrictor factors including D-dimer, CFIII, s-Psel and EN-1, which are deregulated compared to controls, although we found no real differences between type A and B patients. Our findings with ICAM-1 agree with previous studies [28, 29], which reported that during the early phase of AAD deregulation of the production of soluble and intracellular forms of adhesion molecule could result in lower levels of the intracellular form, such as ICAM-1, to compensate the release of circulating mediators, as illustrated in Fig. 2.

The main limitation of this study is the small number of patients and further investigations are obviously needed to increase the power of our results.

## Conclusion

Our findings suggest that hypomagnesemia may be one of the principal causes of AAD onset and development, which can result in different sub-events related to Mg depletion including first of all hypocalcemia and subsequently hypertension, then inflammation and vasoconstriction of the endothelium. Therefore it is plausible that besides the systemic Ca level intra-cellular calcium may increase too in AAD patients during Mg depletion, but this assumption needs to be verified by future analysis to better understand the pathophysiology of AAD and its direct relationship with Mg depletion.

## Abbreviations

AAD: Acute aortic dissection
Ca: Calcium
CFIII: Coagulation factor III
CVD: Cardiovascular disease
ELISA: Enzyme-linked immunosorbent assays
EN-1: Endothelin-1
hCRP: Human high-sensitive C-reactive protein
ICAM-1: Intercellular adhesion molecule 1
IL-1β: Interleukin-1β
IL-6: Interleukin-6
Mg: Magnesium
SD: Standard deviation
s-Psel: Soluble P-selectin
TNF-α: Tumor necrosis factor-α
VSMC: Vascular smooth muscle cells
